# Structure of New Binary and Ternary DNA Polymerase Complexes From Bacteriophage RB69

**DOI:** 10.3389/fmolb.2021.704813

**Published:** 2021-11-18

**Authors:** Jongseo Park, Hyung-Seop Youn, Jun Yop An, Youngjin Lee, Soo Hyun Eom, Jimin Wang

**Affiliations:** ^1^ School of Life Sciences, Gwangju Institute of Science and Technology (GIST), Gwangju, South Korea; ^2^ Steitz Center for Structural Biology, GIST, Gwangju, South Korea; ^3^ BIO R&D Center, Ingredient Business Unit, Daesang Corporation, Gyeonggi-do, Korea; ^4^ Virocure Inc., Seoul, Korea; ^5^ Metabolic Regulation Research Center, Korea Research Institute of BIoscience and Biotechnology (KRIBB), Daejeon, Korea; ^6^ Department of Chemistry, GIST, Gwangju, Korea; ^7^ Department of Molecular Biophysics and Biochemistry, New Haven, CT, United States

**Keywords:** RB69 DNA polymerase, crystal structure, open binary complex, closed ternary complex, single-divalent metal ion–containing complex, replisomal complex

## Abstract

DNA polymerase plays a critical role in passing the genetic information of any living organism to its offspring. DNA polymerase from enterobacteria phage RB69 (RB69pol) has both polymerization and exonuclease activities and has been extensively studied as a model system for B-family DNA polymerases. Many binary and ternary complex structures of RB69pol are known, and they all contain a single polymerase-primer/template (P/T) DNA complex. Here, we report a crystal structure of the exonuclease-deficient RB69pol with the P/T duplex in a dimeric form at a resolution of 2.2 Å. The structure includes one new closed ternary complex with a single divalent metal ion bound and one new open binary complex in the pre-insertion state with a vacant dNTP-binding pocket. These complexes suggest that initial binding of the correct dNTP in the open state is much weaker than expected and that initial binding of the second divalent metal ion in the closed state is also much weaker than measured. Additional conformational changes are required to convert these complexes to high-affinity states. Thus, the measured affinities for the correct incoming dNTP and divalent metal ions are average values from many conformationally distinctive states. Our structure provides new insights into the order of the complex assembly involving two divalent metal ions. The biological relevance of specific interactions observed between one RB69pol and the P/T duplex bound to the second RB69pol observed within this dimeric complex is discussed.

## Introduction

DNA replication at the replication fork is the semi-discontinuous process; one strand is the leading strand, and the other strand is the lagging strand (Hamdan et al., 2009; Yao and O'Donnell, 2008). In the presence of externally supplied primers, a minimal T4 replication model containing only two polymerases (pols), a lagging strand polymerase and a leading polymerase, is capable of forming trombone loops mimicking these in the holo-assembly at the replication fork (Alberts et al., 1983; Noble et al., 2015; Benkovic and Spiering, 2017). However, direct pol–pol interactions are relatively weak (Salinas and Benkovic, 2000), suggesting that coordination of DNA synthesis of both strands may be mediated by other replisomal proteins or/and by a DNA duplex. This study provides evidence for such coordination *via* a primer/template (P/T) DNA duplex.

DNA polymerases are very dynamic, undergo many conformational changes with correct incoming dNTPs, and transverse various distinctive functional states such as pre-insertion or post-insertion (which can be in pre-translocation or post-translocation) according to their interactions with both dNTPs and the P/T duplex (Franklin et al., 2001; Berman et al., 2007). The DNA polymerase from RB69 phage (RB69pol) belongs to the B-family pols and shares a high degree of similarity with human pols (Wang et al., 1997). It contains two activities (polymerase and exonuclease) and is a classic model for studying DNA replication (Wang et al., 1997; Alberts, 2003). RB69pol has been extensively studied both structurally and biochemically with respect to its domain architecture, substrate binding, nucleotide incorporation, and metal coordination in both polymerizing and editing modes (Wang et al., 1997; Shamoo and Steitz, 1999; Franklin et al., 2001; Hogg et al., 2004; Aller et al., 2007; Hogg et al., 2007; Zahn et al., 2007; Hogg et al., 2010; Aller et al., 2011; Xia et al., 2011a; Zahn et al., 2011a; Xia et al., 2011b; Zahn et al., 2011b; Xia et al., 2012b; Xia et al., 2013b). RB69pol has the following distinct domains: the N-terminal, Exonuclease, Palm, Fingers, and Thumb domains, and a functionally important tail for the assembly of the holo-enzyme at the replication fork.

DNA synthesis by DNA pols is catalyzed by two divalent metal ions, A and B (Steitz, 1999). Metal ion A generates the attacking hydroxyl anion of the 3′-nucleotide of the primer strand (*pt*O3′) as a general base, and metal ion B stabilizes the leaving pyrophosphate as an acid in the transition state (TS). Upon binding of correct incoming dNTPs, a large conformational change occurs to close down the fingers domain and to bury the reactants inside the pol for catalysis (Franklin et al., 2001). The apparent binding affinities for both Watson–Crick base-paired dNTPs and divalent metal ions are very high (apparent *K*_d_ ∼50 µM) (Xia et al., 2013a). These apparent values are averaged from many conformational steps. Within the closed ternary complex, many additional hydrogen bonds (with residues of Arg482, Lys660, and Asn564) are formed that are not present in the initial binding of the dNTP in the open complex. Therefore, initial binding would be expected to be much weaker than the final binding step immediately before chemistry, implying that the actual binding affinity of the final substrate complex near the TS could be much higher than the measured average values. The assembly of the replication complex clearly involves many steps, not all of which have been fully characterized structurally, including the initial binding of the first divalent metal ion followed by the second metal ion which is dehydrated inside the closed complex because metal ion B has no water ligand in the final substrate complex. In this study, we capture two new intermediates in a single dimeric structure, one corresponding to an open complex with a vacant dNTP-binding site where the apparent binding affinity of the dNTP in this specific conformational state is likely to be very low, and the second corresponding to a one-metal ion bound closed ternary complex where the binding affinity of the second divalent metal ion should also be very low. Additional conformational changes are required to convert these low-affinity complexes to high-affinity states. Intermediate structures between the classic open binary complex and the fully closed ternary complex have already been reported for other DNA pols, for example, an “ajar” intermediate (Wu and Beese, 2011). Nevertheless, how these intermediates are correlated with the base selectivity of nucleotide incorporation by DNA pols remains poorly understood.

## Materials and Methods

### Protein Overexpression and Purification

The exonuclease-deficient (*exo* ^−^ with the mutations of D222A and D327A) RB69pol variant gene was a gift from Professor William H. Konigsberg (Yale University, New Haven, CT, United States ) and amplified by PCR using a 2,720 thermal cycler (Applied Biosystems). PCR was run in ThermoPol buffer (New England Biolabs) using *Pfu* DNA polymerase (New England Biolabs). The forward and reverse primers of RB69pol RB69pol (5′-CGC GGA TCC ATG AAA GAA TTT TAC TTA AC-3′ and 5′-CCG CTC GAG TCA AAA ATC GAA CAT ATC G-3′) were designed from the nucleotide sequences in GenBank, accession numbers AAP75958.1. The amplified genes and the modified pET28b expression vector were digested using BamHI and XhoI and then ligated into the expression vector, which was used to transform *Escherichia coli* strain BL21 (DE3). The transformed BL21 (DE3) cells were grown overnight at 37°C in 50 ml Luria-Bertani broth containing 50 μg/ml kanamycin (Duchefa Biochemie). Cells were resuspended in 4 L of the same media and grown at 37°C to an OD_600_ of 0.4–0.6. His_6_-tagged recombinant protein was then induced using 0.5 mM isopropyl-β-D-thiogalactoside (Pharmacia) and incubated for 16 h at 20°C. After incubation, cells of 11.7 g were obtained, which contained the overexpressed RB69pol *exo* ^−^. Cells were resuspended in lysis buffer [50 mM sodium phosphate (pH 8.0), 300 mM NaCl, 5 mM imidazole, and 10% (*v*/*v*) glycerol]. The lysate was then produced using an ultrasonic processor (Sonics) and cleared by centrifugation at 1,593 *g* for 1 h. The His_6_-tagged recombinant protein was purified with Ni-NTA chelating agarose resin (Peptron, Korea) by washing with lysis buffer and eluting with 50 mM sodium phosphate (pH 8.0), 300 mM NaCl, and 150 mM imidazole. Tobacco etch virus protease was added into the eluted protein and incubated for 12 h at 4°C to remove the N-terminal His_6_-tag. The protein was further loaded onto a Superdex 200 16/60 column (GE Healthcare) that was pre-equilibrated in 10 mM Tris-HCl (pH 8.0), 50 mM NaCl, 5 mM dithiothreitol (DTT), and 5% glycerol. Fractions containing the RB69pol were pooled and concentrated to 25.8 mg/ml.

### Crystallization

To form a 15-base paired P/T duplex, the primer (5′-GGA​GCG​GAC​TGC​TTA​C-3′) and the template (5′-TCA​AGT​AAG​CAG​TCC​GCT​C-3′) were purchased (Cosmogenetech, Korea) and annealed after mixing in a 1:1 ratio. This P/T duplex was two base pairs longer than one used in an initial study of a single-pol replicating complex of RB69pol (Franklin et al., 2001). RB69pol was then mixed with the P/T duplex at a 1:1.2 stoichiometry to form the RB69pol-P/T complex in 10 mM Tris-HCl (pH 8.0), 50 mM NaCl, 10 mM CaCl_2_, 5 mM dithiothreitol (DTT), and 5% glycerol, followed by the addition of 1 mM 2′-deoxyuridine-5'-(*α,β*-imido) triphosphate (dUpNpp, Jena Bioscience), which was a non-hydrolyzable analog of dTTP, for incubation for 12 h at 4°C. The formed complex was initially screened for crystallization using the sitting-drop vapor-diffusion method in a 96-well INTELLI-PLATE (Art Robbins, Inc.). The final crystallization conditions were obtained *via* optimization from the initial crystallization setup performed using *Natrix* HT (Hampton Research). Crystals were grown at 21°C in 2 μl of the drop containing equal volumes of the protein/DNA solution and the reservoir solution, which was composed of 50 mM sodium cacodylate (pH 5.0), 2–7% (*v*/*v*) isopropanol, 5 mM MgCl_2_, 2 mM NaCl, and 12 mM spermine. The crystals were cryo-protected by transferring them to the reservoir solution supplemented with 30% (*v*/*v*) polyethylene glycol 400 (PEG 400) and flash-frozen in liquid nitrogen for data collection.

### Structure Determination

A complete data set was collected at a resolution of 2.2 Å at 100 K at BL-NW12A of the Photon Factory, Japan. Data were indexed and initially processed using the *HKL-2000* package (Otwinowski and Minor, 1997) and then reprocessed using *iMOSFLM* (Battye et al., 2011) and merged using *AIMLIESS* in the *CCP4i* package (Battye et al., 2011). The crystals of the RB69pol-P/T complexes belonged to the *P*3_2_21 space group and had the unit-cell dimensions of *a* = *b* = 164.44 Å, *c* = 165.60 Å, α = β = 90°, γ = 120°. Assuming one dimeric RB69pol-P/T DNA complex molecule was in an asymmetric unit, the Matthews coefficient was 2.23 Å^3^/Da, which corresponded to a solvent content of 45% (Matthews, 1968). Initial molecular replacement calculations were performed using *Phaser* in the *CCP4* suite (McCoy et al., 2007) using search models derived from the previously reported structure (PDB ID 3nci) after the P/T duplex, the C-terminal tail, and the Fingers domain were removed (Franklin et al., 2001). Afterward, the P/T duplex, the Fingers, the C-terminal tail, dUpNpp, and Ca^2+^ were manually built using *Coot* (Emsley et al., 2010), followed by automated water picking with *ARP/wARP* (Langer et al., 2008). Several steps of manual rebuilding and refinement of this structure were performed using *Coot* and *Refmac5* (Murshudov et al., 2011); the final crystallographic *R* value was 19.3% (*R*_free_ = 24.5%). The statistics for data collection and refinement are summarized in Table 1.

**TABLE 1:** Data processing and model refinement statistics (PDB ID 7f4y).

Data processing statistics	
X-ray source	PF-NW12A
Wavelength (Å)	1.0000
Space group	*P*3_2_21
Unit cell parameters	*a* = *b* = 164.44 Å, *c* = 165.60 Å, α = β = 90°, γ = 120°
Resolution (Å)	65.41–2.20 (2.24–2.20)
Total partial observations	1,635,833
Unique reflections	130,936
Completeness (%)	100.0 (100.0)
*R*_merge_^a^	0.13 (1.86)
R_*PIM*_^b^	0.04 (0.56)
*CC*_1/2_^c^	0.99 (0.54)
Multiplicity	12.4 (12.0)
*I*/σ(*I*)	11.2 (1.8)
**Refinement statistics**	
Resolution (Å)	50.−2.2
Overall *R*_work_ (%)	19.3
Overall *R*_free_ (%)^d^	24.5
R.M.S.D. from ideal geometry	
R.M.S.D. bond lengths (Å)	0.012
R.M.S.D. bond angles (°)	1.55
Mean B-factor (Å^2^)	52.7
Ramachandran statistics	
Most favored (%)	95.2
Generously allowed (%)	4.8
Disallowed (%)	0.0

Values in parentheses are for the highest resolution shell. ^a^ *R*_merge_ = ∑_*hkl*_ ∑_*i*_ |*I*_*i*_(*hkl*) −⟨*I*(*hkl*)⟩|/∑_*hkl*_ ∑_*i*_ *I*_*i*_(*hkl*), where *I*_*i*_(*hkl*) is the intensity of the *i*th observation of reflection *hkl,* and⟨*I*(*hkl*)⟩is an average intensity of reflection (*hkl*). ^b^R_PIM_ is redundancy-corrected merging R-factor. ^c^CC_1/2_ is Pearson correlation coefficient. ^d^ *R*_free_ calculated with 5% cross-validation set.

### Structural Analysis

All structural figures were generated using *PyMOL* version 2.4.0 (Schrödinger LLC). Multiple sequence alignment (see Supporting Materials) was performed using *Clustal X* and visualized using *JalView* (Jeanmougin et al., 1998; Waterhouse et al., 2009). The σ_A_-weighed 2F_o_-F_c_ map was calculated using *phenix.maps* and *phenix.mtz2map* in the CCP4 format (Liebschner et al., 2019). The *PDBePISA* web server was used for the interface analysis (Krissinel and Henrick, 2004).

## Results and Discussion

### The Crystal Structure of the Two Polymerase Complexes From Bacteriophage RB69

**FIGURE 1:**
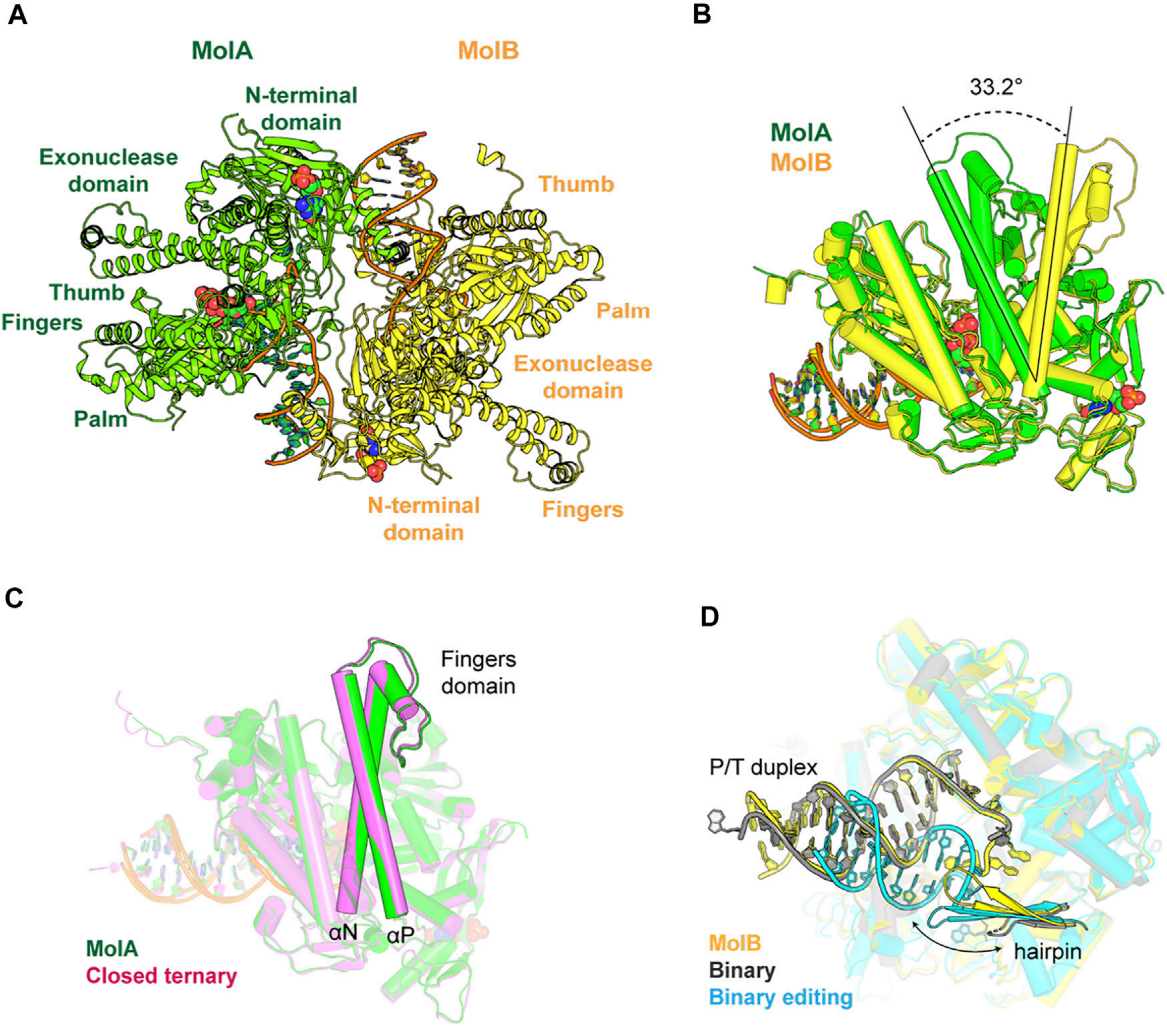
Overall structure of RB69pol-DNA complexes. **(A)** Structures of the ternary complex (MolA, green) and the binary complex (MolB, yellow). Nucleotide molecules are shown as cartoons and spheres. **(B)** Superimposition of ternary and binary complexes and the conformational change of the Fingers domain is shown as the two-sided dashed line. **(C)** Same conformation of the Fingers domains of MolA (green) and a closed ternary complex reported previously (magenta, PDB ID 1ig9). **(D)** Different topologies of the P/T duplex and hairpins of MolB (yellow) and other binary complexes (PDB ID 3l8b, 1clq).

We crystallized the exonuclease-deficient RB69pol complex with the P/T duplex and determined its structure at a resolution of 2.2 Å with a free R-factor of about 24.5% (PDB ID 7f4y) (Table 1). Two RB69pol-P/T complexes are present in one asymmetric unit (Figure 1A). The N-terminal domain (NTD) of RB69pol and the part of the P/T duplex away from the active site participate in the dimerization of the two complexes. One RB69pol-P/T complex (MolA) is identified as a new closed ternary complex with dUpNpp, which was similar to many other known ternary complexes of this enzyme. However, this complex contained only one divalent metal ion. The other complex (MolB) is identified as a new open binary RB69pol-P/T complex in the pre-insertion state but without dUpNpp bound and with a vacant dNTP-binding pocket. The major differences of the two complexes are rotations (approximately 33.2°) of the Fingers domain, as previously reported (Figure 1B) (Franklin et al., 2001).

### A New Closed Ternary Complex With Single Divalent Metal Ion Bound

**FIGURE 2:**
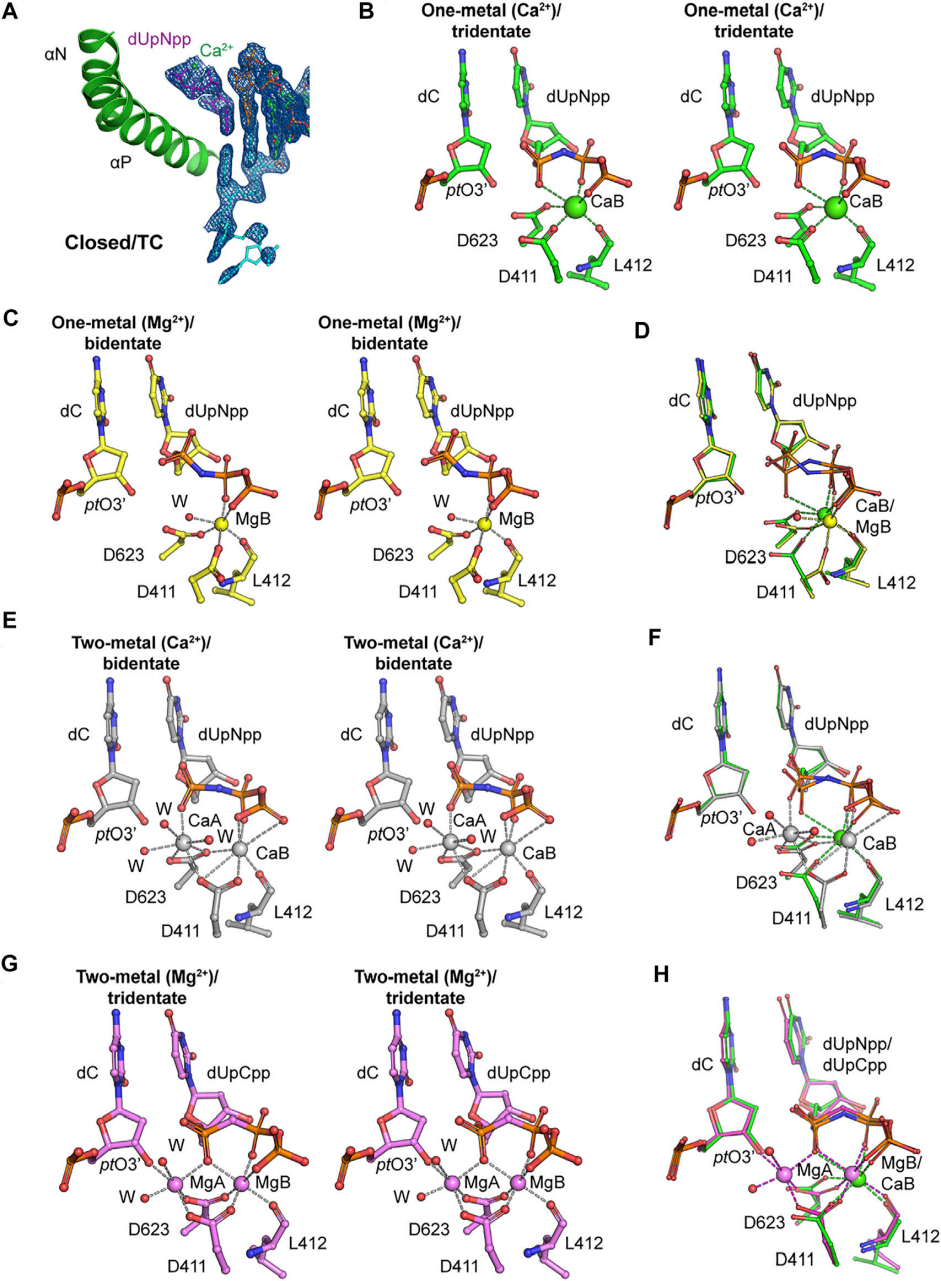
Metal coordination in the closed ternary complexes. **(A)** Closed ternary complex (MolA) containing one divalent metal ion bound to the triphosphate moiety instead of the two observed previously. Incoming dNTP (dUpNpp), primer, and template are in magenta, orange, and cyan, respectively. The σ_A_-weighted 2*F*_obs_-*F*_calc_ electron density maps are contoured at 1.5 σ. **(B)** Stereodiagram of tridentate Ca^2+^ coordination in the one–metal ion replication complex (MolA) with octahedral geometry. The Ca^2+^ ion is in green, and Ca^2+^ coordination is marked with dashed lines. **(C)** Stereodiagram of bidentate Mg^2+^ coordination in the one–metal ion replication complex (PDB ID 3si6) with octahedral geometry. The Mg^2+^ ion is in yellow, and Mg^2+^ coordination is marked with dashed lines. **(D)** Superimposed representation of Ca^2+^ or Mg^2+^ coordination complexes of the one–metal ion with tridentate coordination (MolA, green) and with bidentate coordination (yellow). The metal coordination is in green or yellow in tridentate or bidentate coordination complexes, respectively. **(E)** Stereodiagram of bidentate Ca^2+^ coordination in the two–metal ion replication complex (PDB ID 3uiq) with pentagonal bipyramidal geometries for sites A (CaA) and B (CaB), and CaA does not bind to *pt* O 3’. Ca^2+^ ions and water molecules are in gray and red, respectively. **(F)** Superimposed representation of Ca^2+^ coordination complexes of the one–metal ion with tridentate coordination (MolA, green) and two–metal ion with bidentate coordination (gray). The Ca^2+^ coordination is in green or gray in one–metal ion or two–metal ion replication complexes, respectively. **(G)** Stereodiagram of tridentate Mg^2+^ coordination in the two–metal ion replication complex (PDB ID 3spy) with octahedral geometries for sites A (MgA) and B (MgB), and the MgA interacts with *pt* O 3’. Mg^2+^ ions and water molecules are in magenta and red, respectively. **(H)** Superimposed representation of metal coordination complexes of a Ca^2+^-bound one–metal ion with tridentate coordination (MolA, green) and Mg^2+^ bound two–metal ion with bidentate coordination (magenta). The metal coordination is in green or magenta in Ca^2+^-bound one–metal ion or Mg^2+^-bound two–metal ion replication complexes, respectively.

In the MolA structure, the nascent Watson–Crick base pair dA/dUpNpp was well defined in electron density maps, as were all the 5’ overhanging nucleotide residues of the template strand (Figure 2A). The conformation of the Fingers domain in this structure was essentially the same as the one in the closed ternary complex reported previously with a root mean square deviation of 0.34 Å for all Cα coordinates (Figure 1C) (Franklin et al., 2001), representing a closed ternary complex before chemistry. Although the crystals were grown in the presence of 10 mM CaCl_2_, the standard cryoprotectant solution commonly used for the RB69pol-P/T complex crystals replaced 10 mM Ca^2+^ ions with 5 mM Mg^2+^ ions, which could eliminate any weakly bound divalent metal ion. Indeed, we observed that it had only a single Ca^2+^ ion bound at the B site but no Ca^2+^ ion at the A site. Thus, there was a significant difference between this complex and the previously reported structures that often contained two catalytic divalent metal ions at both the B and A sites (Franklin et al., 2001; Xia et al., 2012a; Xia et al., 2013a). This observation suggests that the binding affinity of the divalent metal ion at the A site should be much weaker in this specific conformation than the affinity observed in the previous monomeric RB69pol-P/T complex and the measured average value in steady-state kinetics (Xia et al., 2013a).

This structure adds to a new variation on the closed ternary complexes of RB69pol with respect to both the number of divalent metal ions and the conformations of the triphosphate moiety of incoming dNTP. Similar to the classic ternary complex, the B site in MolA of this structure was coordinated by the catalytic carboxylates Asp411 and Asp623 and by the backbone carbonyl of Leu412; in turn, it binds the triphosphate moiety of incoming dNTP with α,β,γ-tridentate coordination (Figure 2B). Previously, a single Mg^2+^ ion bound in the closed ternary complex at the B site was reported, which forms a β,γ-bidentate coordination (Figure 2C) (PDB ID 3si6) (Xia et al., 2011a). Comparison of these two structures suggests that a conversion of the bidentate to tridentate coordination is likely to be a necessary step because Mg^2+^ ions can typically neutralize only two negative charges of two phosphate groups by forming only the bidentate coordination of the triphosphate moiety of a free dNTP in solution, but not with three phosphates simultaneously (Figure 2D). This conversion requires complete dehydration of the B metal ion so that it can be fully buried inside the complex. When the non-exchangeable Rh^3+^/dNTP complex was used in kinetical studies, this step would be kinetically invisible because these studies revealed only the kinetic properties of the binding of the A metal ion (Bakhtina et al., 2005; Lee et al., 2009; Wang et al., 2009).

Another conformation of β,γ,-bidentate Ca^2+^ coordination was observed previously in an RB69pol ternary complex (Figure 2E) (PDB ID 3uiq) (Xia et al., 2012b). However, that complex had two Ca^2+^ ions bound, one at each of the B and A metal ions (Figures 2E,F). Therefore, the order of the bidentate-to-tridentate conversion and the binding of metal ion A (the second site) may be stochastically independent processes, providing a mechanism of allostery and regulation of binding affinities on the kinetic pathway of dNMP incorporation. Both processes should be essential for catalysis because with β,γ,-bidentate coordination to metal ion B (the first site), the α-phosphate of the triphosphate moiety of the incoming dNTP would be free to move in an arc, and because without the binding of metal ion A, the 3′-hydroxyl of the terminal nucleotide of the primer strand (*pt*O3′) could not be brought close enough to the freely moving α-phosphate of the dNTP. In the catalytically competent complex of RB69pol (Xia et al., 2011a), metal ion A binds both the α-phosphate of dNTP and the *pt*O3’, bringing them closer together for chemistry (Figure 2G) (PDB ID 3spy) (Xia et al., 2011a).

Without considering the stochastic kinetic schemes discussed above, kinetic studies may not be sufficient to explain the kinetic behaviors of nucleotide selectivity for dNMP incorporation by DNA pols. In fact, allosteric regulation of binding affinities of metal ions in the two metal-ion sites and the conformational changes associated may play important roles in base selectivity. With the correct incoming dNTP, these two processes are likely to be highly cooperative, that is, the binding of the second divalent metal ion could accelerate the bidentate-to-tridentate conversion of the triphosphate moiety of the incoming dNTP. Simultaneously, this conversion could also stabilize the binding of the second divalent metal ion. However, with an incorrect incoming dNTP, these two processes may not be cooperative, that is, the binding of the second divalent metal ion may prevent the bidentate-to-tridentate conversion by transiently stabilizing the bidentate conformation of the incorrect dNTP, which would eventually be rejected. Simultaneously, the bidentate-to-tridentate conversion may destabilize the binding of the second divalent metal ion because distorted Watson–Crick base-pairing geometry could prevent the tridentate moiety from properly fitting into its putative binding pocket when both metal ions are present. Without both metal ions, chemistry cannot occur, and the incorrect dNTP would eventually be rejected. Based on this analysis, we conclude that our new closed ternary complex of MolA is not the catalytically competent complex but rather a complex on its way to formation of the pre-chemistry ternary complex immediately before catalysis (Figure 2H).

### New Open Binary Complex

**FIGURE 3:**
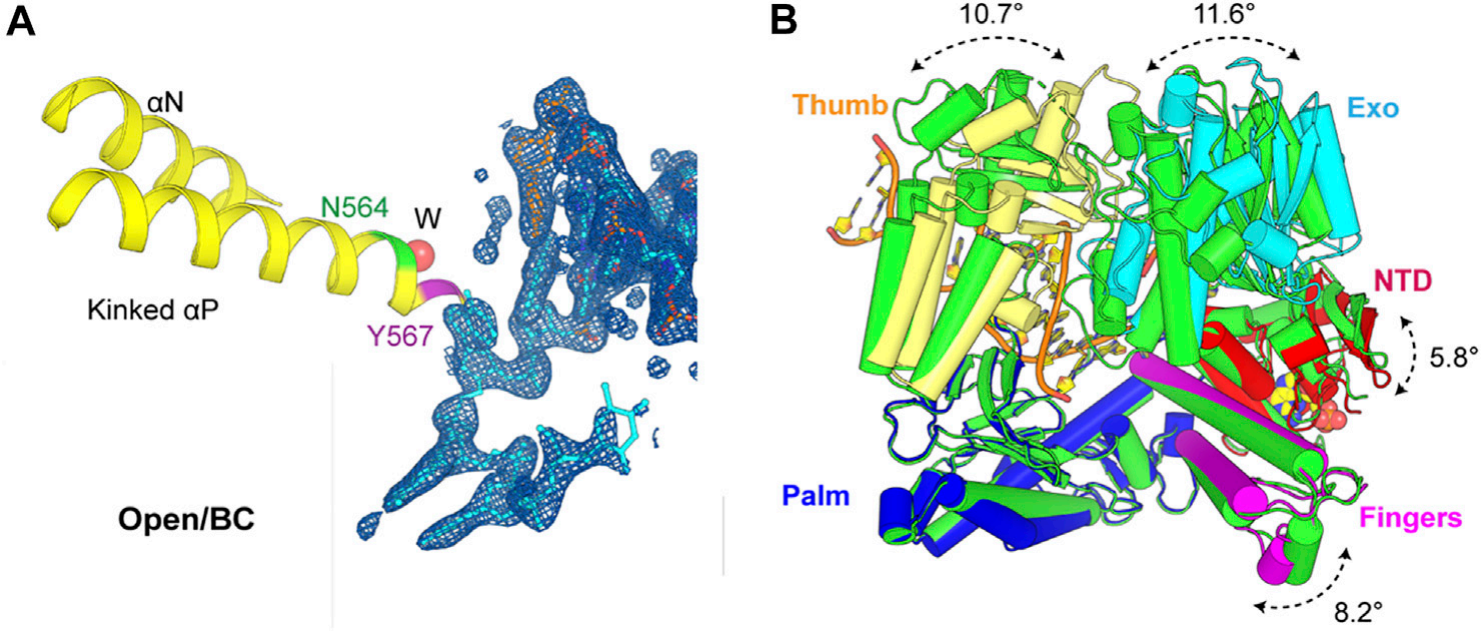
Active site of the open binary complex (MolB) and overall structure comparison with apo structure reported previously. **(A)** Open binary complex (MolB) containing a vacant dNTP-binding site with one ordered water molecule (W) inserted into the backbone of the kinked helix *p* instead of the two observed in the apo structure. Asn564 and Tyr567 are in green and magenta, respectively. The 2*F*_obs_-*F*_calc_ electron density maps are contoured at 1.5 σ. **(B)** Exo (cyan), Thumb (yellow), Fingers (magenta), and NT (red) domains rotate relative to the Palm (blue) domain from this binary complex compared to the apo (green) structure by 11.6°, 10.7°, 8.2°, and 5.8°, respectively.

In the open binary complex of MolB, the penultimate base pair was also well defined in electron density maps alongside all the 5’ overhanging nucleotide residues of the template strand (Figure 3A). In this binary complex, the P/T duplex was completely superimposable with the closed ternary complex, representing a pre-insertion complex where the nascent base pair–binding pocket was partially formed and ready for binding of the next incoming dNTP. However, the next templating base was well ordered but remained in a non-stacking geometry with the proceeding base pair. The base of this nucleotide formed hydrogen bond interactions with the enzyme through ordered water molecules and remained highly hydrated, as seen in Supplementary Figure S1.

This open complex differed from all the previously reported binary complexes that had non–Watson–Crick geometry for the nascent base pair or non-stackable templating nucleotide residues (Figure 1D) (Shamoo and Steitz, 1999; Aller et al., 2010). It also differed from the conformation of the apo structure (PDB ID 1ih7) (Franklin et al., 2001). In this binary complex, there was only one ordered water molecule placed between the Asn564 and the Tyr567 backbone in the kinked helix *p*, whereas there were two ordered water molecules observed in the open conformation of the apo form. The Exo, Thumb, Fingers, and N-terminal domains (NTD) rotated relative to the Palm domain from this binary complex compared to the apo structure by 11.6°, 10.7°, 8.2°, and 5.8°, respectively (Figure 3B). Collectively, the open binary complex showed not only a difference in the rotation of the Fingers domain but also in the rotation of the template base opposite the incoming dNTP compared to the closed ternary complex. This new open complex adopted a pre-insertion form, which was not observed previously.

### Unique Nucleotide Binding Pocket and Conformational Change by GMP at the N-Terminal Domains

**FIGURE 4:**
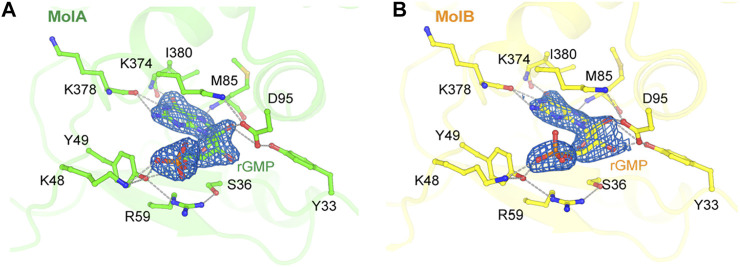
Nucleotide binding site in the N-terminal domains of MolA **(A)** and MolB **(B)**. The σ_A_-weighted 2*F*_obs_-*F*_calc_ electron density maps are contoured at 1.5 σ. Hydrogen bonds are displayed as dashed lines.

There was a conserved small cavity in the NTDs of each RB69pol that tightly bound GMP (Figure 4). The surface area buried between the bound GMP and RB69pol was ∼331 Å^2^. This binding involved eight hydrogen bonds with the Tyr49/Asp95 side chains and the backbones of Met85/Lys378/Ile380 and one salt-bridge interaction with the Lys48 side chains. This nucleotide most likely came from copurification because it was never added during purification.

The binding of GMP at this location was also observed previously in the apo structure of RB69pol and the key interaction pattern was conserved with those of our new structure, but it was not present in the isolated NTD/Exo domain structure of T4 DNA polymerase (T4pol) (Wang et al., 1997; Wang et al., 1996). In all previous P/T ternary complexes of RB69pol, this pocket was occupied by the overhanging 5′-dG template nucleotide of a neighboring molecule (Supplementary Figure S2) (Franklin et al., 2001; Aller et al., 2007). Comparison of the RB69pol/T4pol structures with and without GMP (namely, in the truncated T4 exonuclease domain) revealed a large GMP-dependent NTD conformational change, including the re-orientation of helix αA by 90° (Supplementary Figure S3).

### GMP-Dependent Conformations of N-Terminal Domains for Formation of the Two-P/T Two-Pol Complex

**FIGURE 5:**
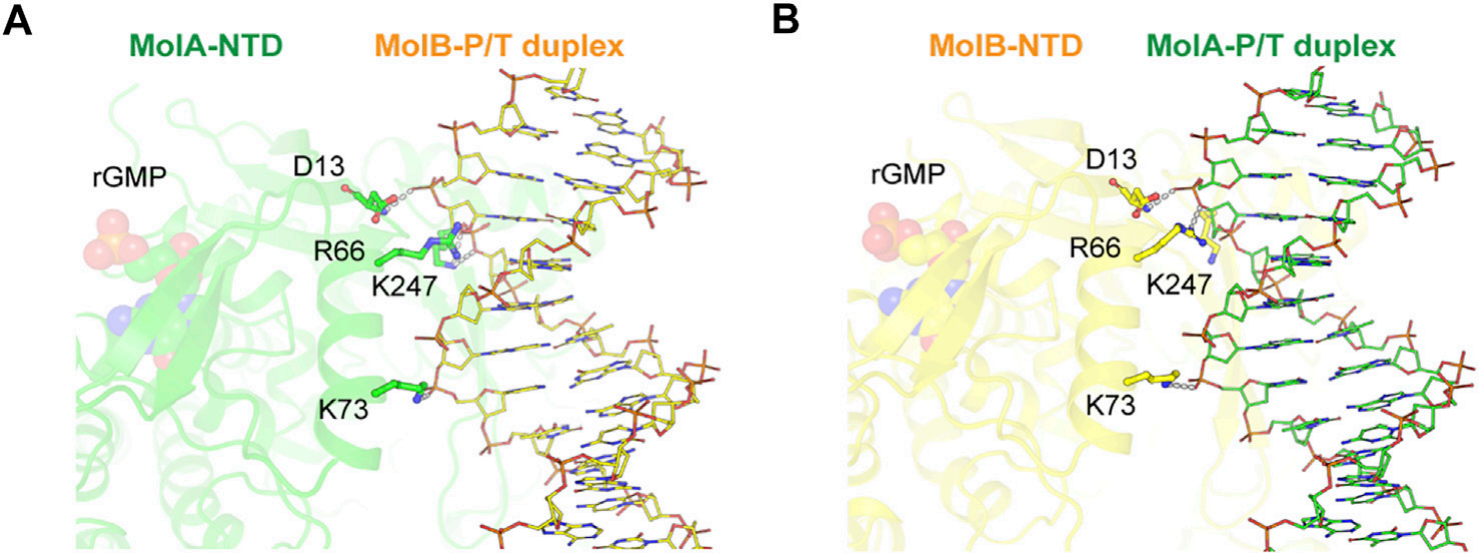
Inter-complex interaction between ternary (MolA, green) and binary (MolB, yellow) complexes. Detailed views of interactions for the NTD (MolA) with the P/T duplex (MolB) **(A)** and the NTD (MolB) with the P/T duplex (MolA) **(B)** complexes. Nucleotides (rGMP) bound to the N-terminal domain and DNA helices are shown as spheres and sticks, respectively. Hydrogen bonds between the N-terminal domain in one subunit and the DNA strand in the other subunit are displayed as dashed lines.

Each NTD of two RB69pol molecules interacted with the part of the P/T duplex away from the pol active site (Figure 5). This interaction included the Asp13, Arg66, Lys73, and Lys247 side chains. Among them, Arg66 was highly conserved and protruded into the minor groove of the P/T duplex bound in the second opposing RB69pol complex within the dimer (Supplementary Figure S4). As the two complexes were in two different conformations, inter-complex interactions were also slightly different in interface areas buried between the NTD of MolA and the P/T duplex of MolB (385.5 Å^2^) and between the NTD of MolB and the P/T duplex of MolA (291.6 Å^2^). It appears that the interactions of the closed RB69pol with the P/T duplex of the open complex were stronger than those of the open RB69pol with the P/T duplex of the closed RB69pol complex (Figures 5A,B).

Given the observation that the conformation of the NTD was GMP-dependent, we investigated how this might affect the formation of the dimeric complex. Using computer modeling, we observed that only the GMP-bound conformation could form this dimer and that the conformation of the NTD without GMP could result in severe stereochemical clashes in this dimer (Supplementary Figure S3). Interestingly, the RB69pol NTD had the common βαββαβ topology found in many oligonucleotide-binding and RNA-binding proteins (Burd and Dreyfuss, 1994; Liu et al., 2006). Assembly and disassembly of a minimal replication complex containing both leading and lagging replication complexes were extensively studied in the T4 system, including the use of the two-hybrid system (Salinas and Benkovic, 2000; Benkovic and Spiering, 2017). Given the sequence identity of 61% between RB69pol and T4pol, it is likely that the dimerization observed here is relevant in both systems, but how it is controlled by the binding and release of GMP in this pocket requires additional studies.

### Implications of New Conformations on the Dynamics of DNA Pols and on Base Selectivity

It was unexpected that a partially open P/T complex of RB69pol was captured under the conditions favorable for formation of the closed replication complex. On the other hand, the lengths of the P/T duplex used in earlier studies were too short, so they may have precluded the formation of this dimeric complex. In fact, the optimized length of the P/T duplex used in kinetic studies and then in earlier crystallization attempts consisted of 13 base pairs because longer P/T duplexes gave complex kinetic results, likely involving two DNA pol complexes binding at both ends of the same DNA duplex (Franklin et al., 2001). Therefore, the dimeric complex with the longer 16-base pair P/T duplex reported here might be more biologically relevant. The structures of both conformations simultaneously captured in our dimeric complex represent the new stable intermediates that were not previously known, and they are clearly involved in forming the catalytically competent complex (Supplementary Figure S5). Our observation also indicated that the fractions of these two conformations of P/T complexes in the population in solution were significant and that they should have similar free energies. Our findings remind us of the fact that other stable intermediates may also exist but remain hidden from us but may be kinetically important for base selectivity.

Our new partially open complex provides new insights into the initial steps in the assembly of the replication complex (Supplementary Figure S5). An initial Watson–Crick base pair is formed between the templating base and the incoming dNTP while the templating nucleobase remains partially hydrated and the Fingers domain remains open and is gradually converted to the partially closed state. Continuous closing of the Fingers domain requires cooperation of binding of two divalent metal ions as discussed above. Simultaneously, dehydration of the nascent base pair occurred alongside closing motions of RB69pol and stacking interactions of base pairs. With the correct incoming dNTP, this process can be cooperatively completed. However, with an incorrect incoming dNTP, full dehydration of the incorrect base pair would be blocked and the binding of two divalent metal ions cannot be accomplished. As a result, an incorrect dNTP would be rejected.

## Data Availability

The dataset presented in in this study can be found in online repository RCSB PDB with the accession number 7f4y (https://www.rcsb.org/structure/7F4Y).
