# Supplementary material for: Structure of New Binary and Ternary DNA Polymerase Complexes From Bacteriophage RB69
Source: Front Mol Biosci. 2021 Nov 18;8:704813. doi: 10.3389/fmolb.2021.704813 (PMC8639217; doi:10.3389/fmolb.2021.704813)
Supplement: Supplementary file 1 [file Presentation1.pdf]

Supplementary Material

Supplementary Figures

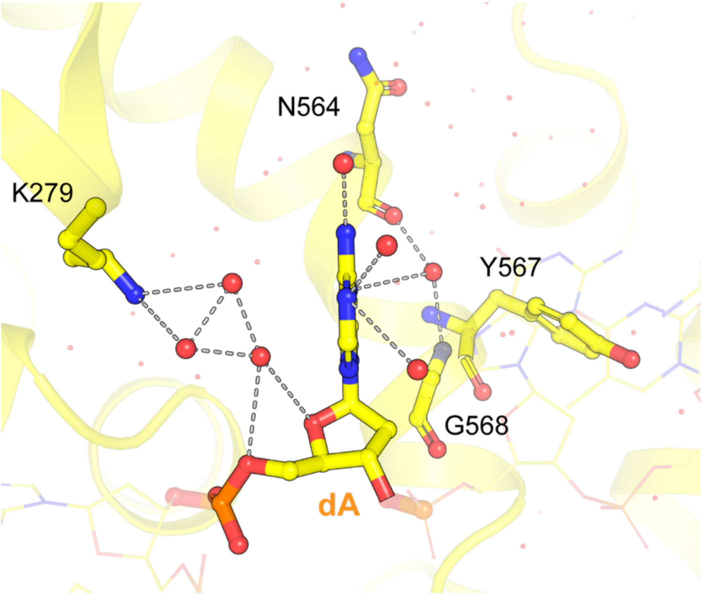

Supplementary Figure 1. Interactions of hydrated nucleotide in MolB with enzyme via ordered water molecules. Water molecules are marked as red spheres. Hydrogen bonds are marked as dashed lines in grey.

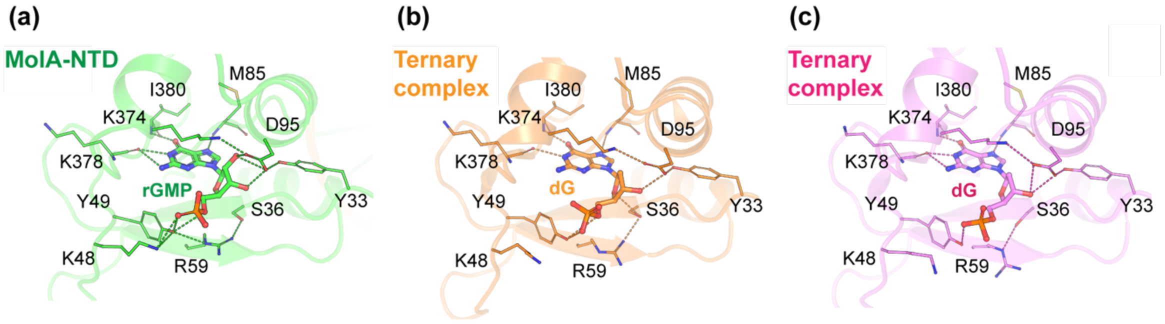

Supplementary Figure 2. Detailed view of the ligand binding site of RB69pol N-terminal domain (NTD). (a) The ligand binding site of NTD in MolA occupied with rGMP. (b) The ligand binding site of NTD in previous determined ternary complex (PDB ID 2dy4) structure occupied with dG. (c) The ligand binding site of NTD in previous determined ternary complex structure (PDB ID 1ig9) occupied with dG.

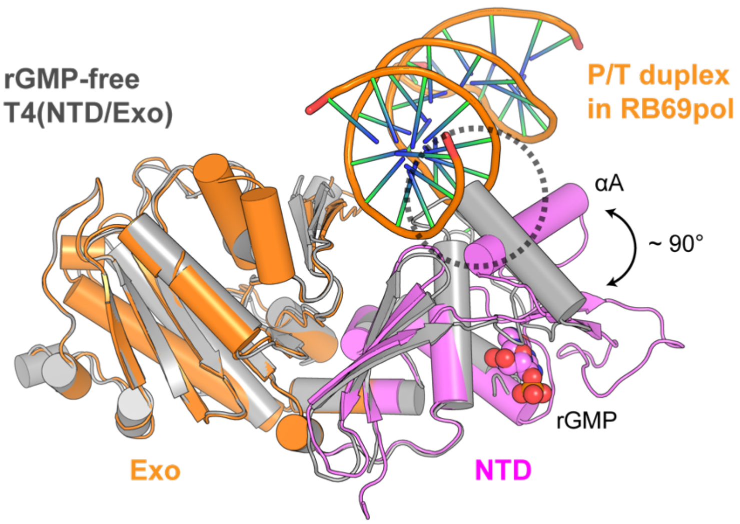

Supplementary Figure 3. Superimposition of rGMP-free T4 NTD/Exo crystal structure (PDB ID 1noy; Grey) with RB69pol-P/T duplex (PDB ID 7f4y; Exo: Orange; NTD: Violet). This comparison shows large conformational changes near the GMP-binding pocket, including the reorientation of helix αA by nearly 90°. The black circle indicates potential steric clashes of GMP-free conformation to the P/T duplex after conformational changes.

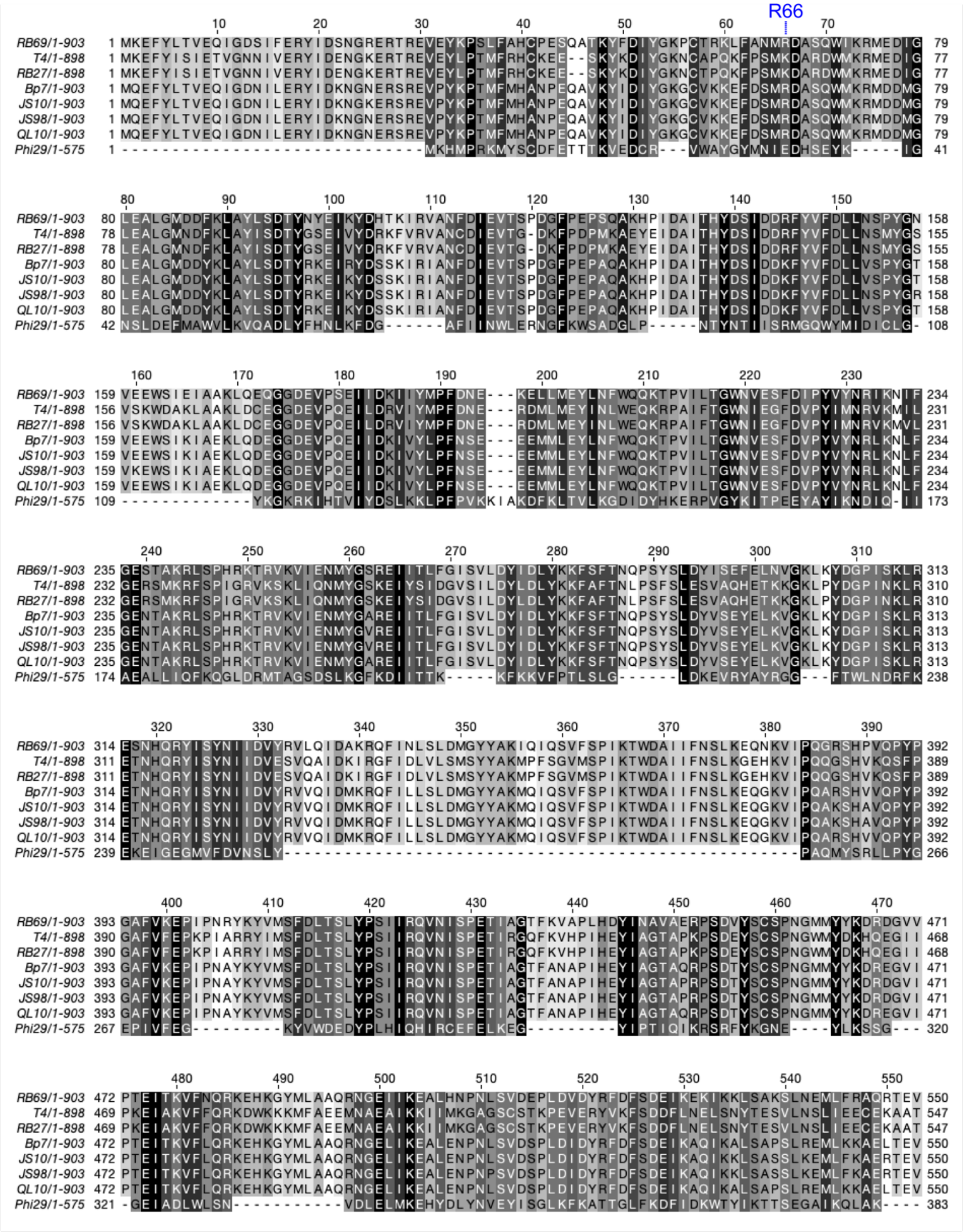
R66
RB69/1-903
T4/1-898
RB27/1-898
Bp7/1-903
JS10/1-903
JS98/1-903
QL10/1-903
Phi29/1-575

(continue)

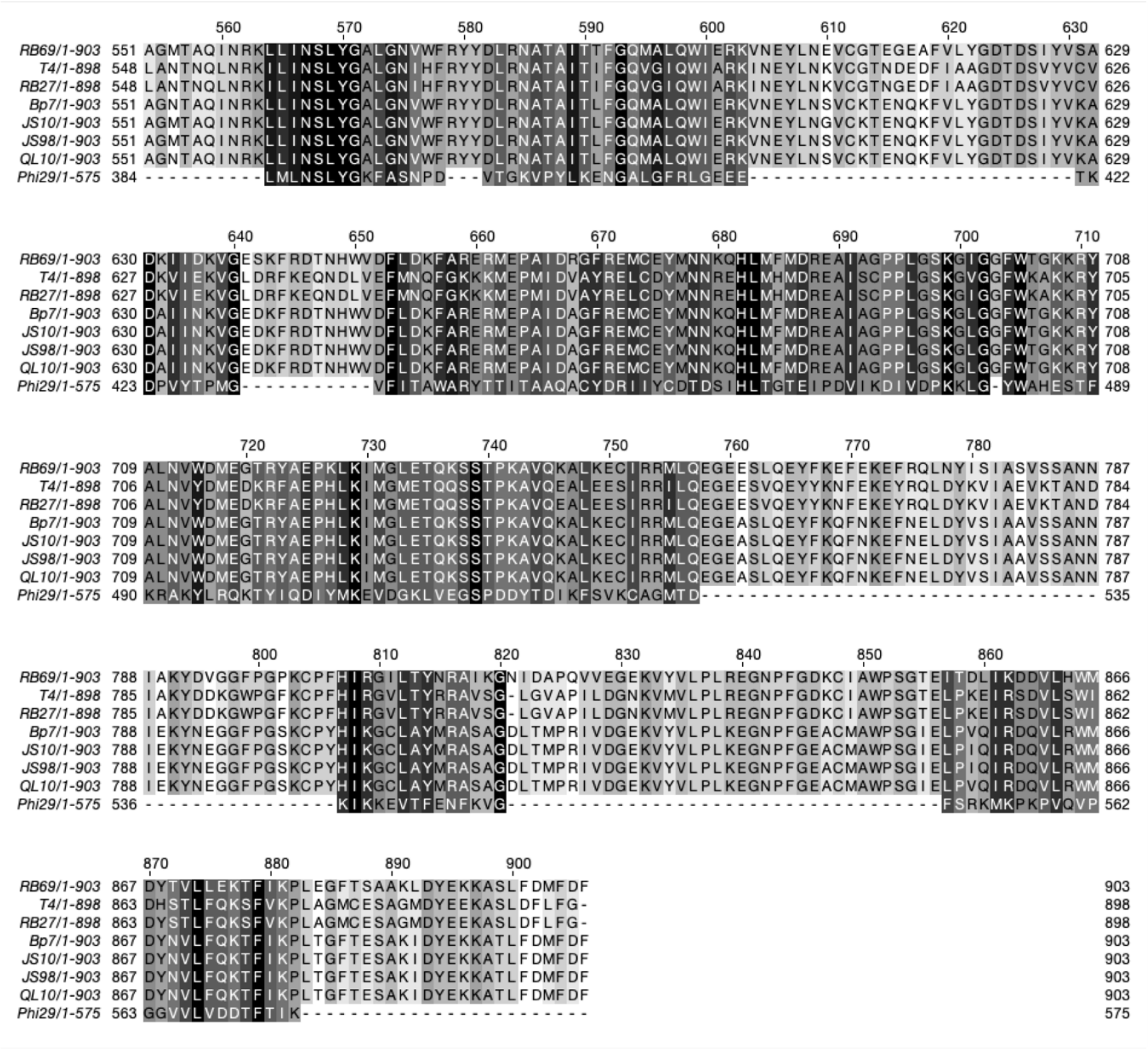

Supplementary Figure 4. Multiple sequence alignment of DNA polymerase from bacteriophages.

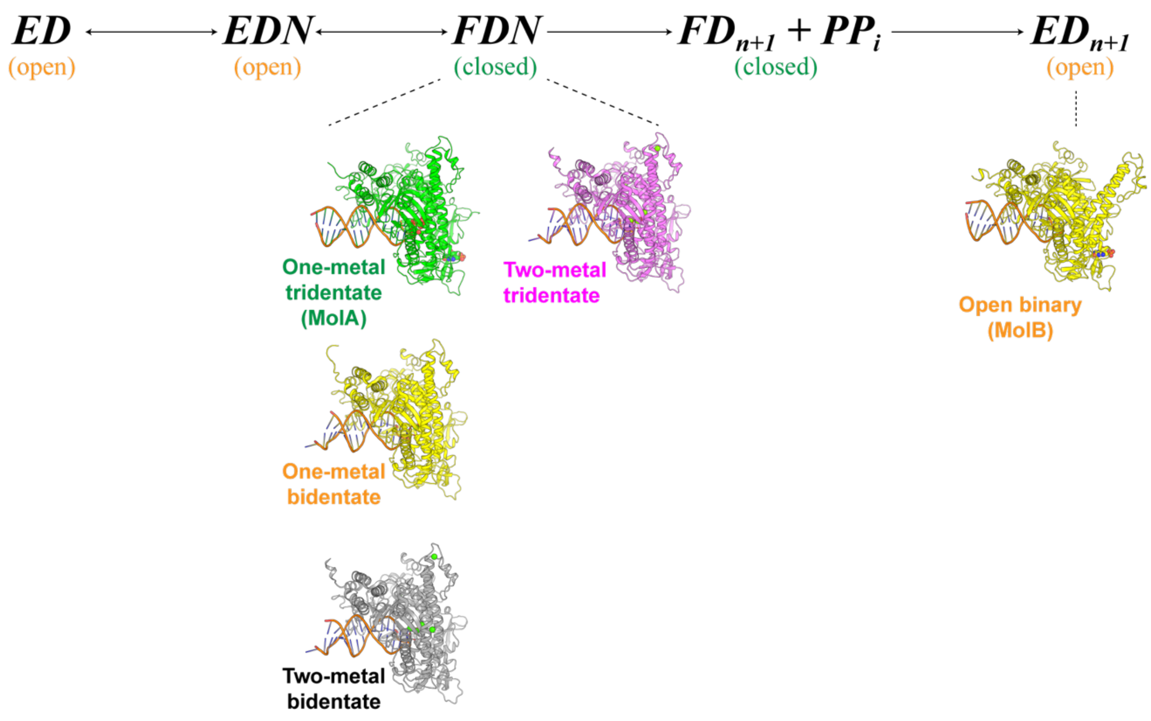

Supplementary Figure 5. Schematic representation of RB69pol catalytic process with several previous structures (PDB ID 3si6, 3uiq, 3spy) (E: RB69pol; D_n: DNA; F: RB69pol in closed conformation; N: dNTP).
